# Diabetes, even newly defined by HbA1c testing, is associated with an increased risk of in-hospital death in adults with COVID-19

**DOI:** 10.1186/s12902-021-00717-6

**Published:** 2021-03-26

**Authors:** Ye Liu, Ran Lu, Junhong Wang, Qin Cheng, Ruitao Zhang, Shuisheng Zhang, Yunyi Le, Haining Wang, Wenhua Xiao, Hongwei Gao, Lin Zeng, Tianpei Hong

**Affiliations:** 1 Department of Endocrinology and Metabolism, Peking University Third Hospital, 49 North Garden Road, Haidian District, Beijing, 100191 P.R. China; 2 Department of Emergency, Peking University Third Hospital, Beijing, 100191 P.R. China; 3 Department of Pulmonary and Critical Care Medicine, Peking University Third Hospital, Beijing, 100191 P.R. China; 4 Department of Cardiology, Peking University Third Hospital, Beijing, 100191 P.R. China; 5 Department of General Surgery, Peking University Third Hospital, Beijing, 100191 P.R. China; 6 Clinical Epidemiology Research Center, Peking University Third Hospital, Beijing, 100191 P.R. China

**Keywords:** Coronavirus disease 2019, Undiagnosed diabetes, Hemoglobin A1c, Mortality

## Abstract

**Background:**

Diabetes is associated with poor coronavirus disease 2019 (COVID-19) outcomes. However, little is known on the impact of undiagnosed diabetes in the COVID-19 population. We investigated whether diabetes, particularly undiagnosed diabetes, was associated with an increased risk of death from COVID-19.

**Methods:**

This retrospective study identified adult patients with COVID-19 admitted to Tongji Hospital (Wuhan) from January 28 to April 4, 2020. Diabetes was determined using patients’ past history (diagnosed) or was newly defined if the hemoglobin A1c (HbA1c) level at admission was ≥6.5% (48 mmol/mol) (undiagnosed). The in-hospital mortality rate and survival probability were compared between the non-diabetes and diabetes (overall, diagnosed, and undiagnosed diabetes) groups. Risk factors of mortality were explored using Cox regression analysis.

**Results:**

Of 373 patients, 233 were included in the final analysis, among whom 80 (34.3%) had diabetes: 44 (55.0%) reported a diabetes history, and 36 (45.0%) were newly defined as having undiagnosed diabetes by HbA1c testing at admission. Compared with the non-diabetes group, the overall diabetes group had a significantly increased mortality rate (22.5% vs. 5.9%, *p* <  0.001). Moreover, the overall, diagnosed, and undiagnosed diabetes groups displayed lower survival probability in the Kaplan-Meier survival analysis (all *p* <  0.01). Using multivariate Cox regression, diabetes, age, quick sequential organ failure assessment score, and D-dimer ≥1.0 μg/mL were identified as independent risk factors for in-hospital death in patients with COVID-19.

**Conclusions:**

The prevalence of undiagnosed pre-existing diabetes among patients with COVID-19 is high in China. Diabetes, even newly defined by HbA1c testing at admission, is associated with increased mortality in patients with COVID-19. Screening for undiagnosed diabetes by HbA1c measurement should be considered in adult Chinese inpatients with COVID-19.

**Supplementary Information:**

The online version contains supplementary material available at 10.1186/s12902-021-00717-6.

## Background

Coronavirus disease (COVID-19), caused by severe acute respiratory syndrome coronavirus 2 (SARS-CoV-2) [1], has spread worldwide, resulting in more than 100 million confirmed infections and over two million deaths as of 1 March, 2021 (see https://covid19.who.int) [2].

The reported mortality rate for hospitalised patients with COVID-19 ranges from 1.4 to 22.5%, which may be due to different characteristics of patient populations, such as age, comorbidities, and the availability of medical resources [3–6]. Studies have shown that elderly patients with underlying comorbidities are at a greater risk of poor outcomes [7–9]. In particular, several studies have highlighted the association between diabetes and poor COVID-19 prognosis. Diabetes is a common comorbidity, and more patients with severe cases of COVID-19 have diabetes than patients with mild symptoms [8, 10–12]. Patients with diabetes also have a higher mortality rate than those without diabetes [13–15]. However, the diagnostic rate of diabetes is currently low, particularly in China [16], leaving many patients undiagnosed and untreated. There is little information on the prevalence of undiagnosed diabetes in the COVID-19 population and whether undiagnosed diabetes is associated with an increased risk of death from COVID-19.

In this retrospective observational study, we described the prevalence of diabetes, including previously diagnosed and undiagnosed diabetes, in hospitalised patients with COVID-19 at a tertiary medical centre in Wuhan, China. Moreover, we investigated whether diabetes, particularly undiagnosed diabetes, was associated with an increased risk of in-hospital death in patients with COVID-19.

## Methods

### Study design and population

**Fig. 1:**
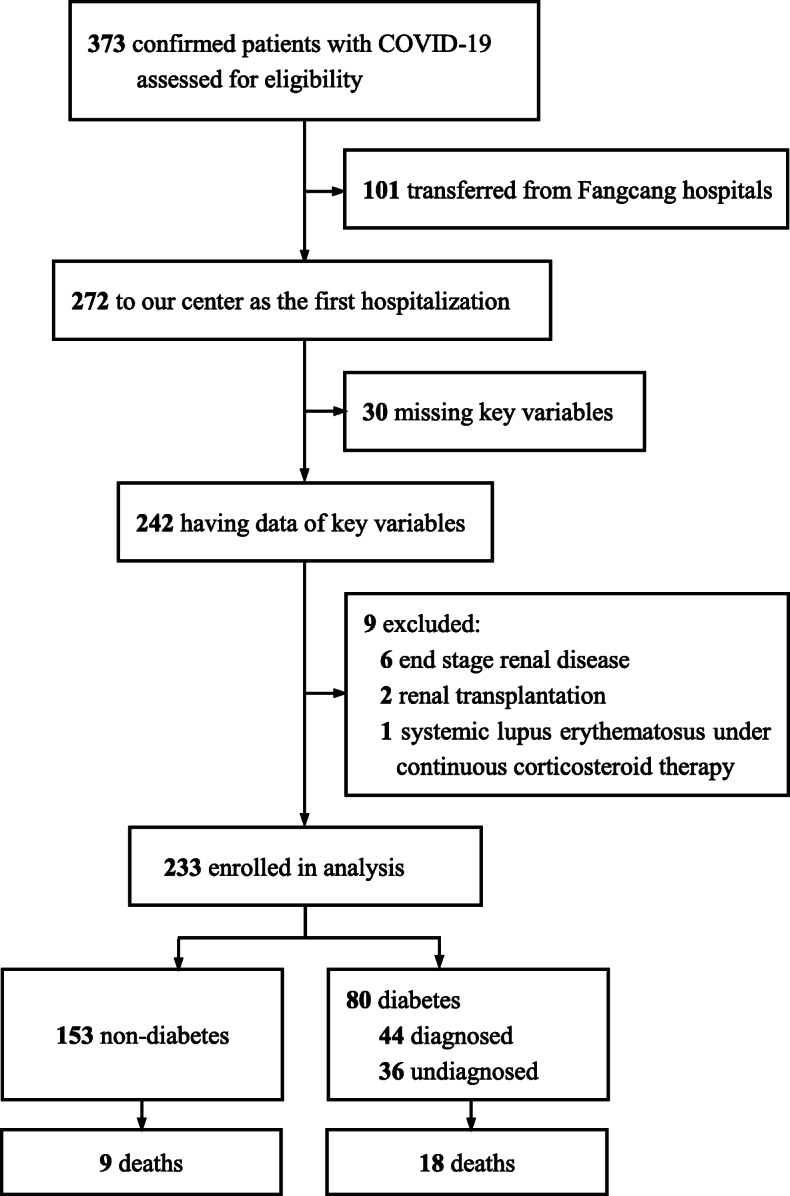
Flow diagram

This retrospective study aimed to investigate the impact of diabetes on the prognosis of COVID-19. We screened all adult patients with a confirmed diagnosis of COVID-19 who were admitted to the COVID-19 wards at Zhongfaxincheng campus of Tongji Hospital in Wuhan, China, from January 28, 2020 to April 4, 2020. In Wuhan, critical, severe, and most moderate patients with COVID-19 were directly admitted to tertiary medical centres such as our institution. Mild patients and a few moderate patients were treated in Fangcang temporary shelter hospitals [17]. If the disease progressed, patients were transferred to tertiary medical centres for further treatment. We excluded transferred patients from Fangcang hospitals to eliminate bias associated with pre-admission treatments. We also excluded patients who lacked records of medical history, vital signs and routine blood test data, and those who had other serious comorbidities (end-stage renal disease or diseases requiring corticosteroid or immunosuppressant therapy) (Fig. 1). All the paitents were followed up to their discharge or in-hospital death.

This study was approved by the Ethics Commission of Peking University Third Hospital (IRB 00006761-M2020060).

### Definition of COVID-19, disease severity and diabetes

The diagnosis of COVID-19 was confirmed if a suspect case with at least two clinical manifestations of COVID-19 was tested positively for SARS-CoV-2 using quantitative polymerase chain reaction assays of nasopharyngeal samples, according to the guideline for COVID-19 issued by the Chinese National Health Committee (version seven). Disease severity classification and treatment protocol were also based on this guideline [18]. Diabetes was determined based on self-reported diabetes history. If patients denied having a history of diabetes and their hemoglobin A1c (HbA1c) levels at admission were ≥ 6.5% (48 mmol/mol) without hemoglobinopathy, they were established to have diabetes.

### Data abstraction

Using a standardised data collection form, the epidemiological records, demographic data, clinical manifestations, laboratory findings, treatment, and outcome data of patients with COVID-19 were extracted from electronic medical records. All data were collected as of April 4, 2020 and were independently checked by two physicians, and a third researcher adjudicated any difference in interpretation between the two physicians.

Quick sequential organ failure assessment (qSOFA) scores were calculated based on systolic blood pressure, respiratory rate in room air, and mental status at admission [19]. Laboratory findings included first in-hospital routine blood test, liver and kidney function test, fasting plasma glucose and HbA1c levels, coagulation profile, and inflammatory markers. HbA1c testing was performed by high-performance liquid chromatography (HA-8180, Arkray; Kyoto, Japan).

### Statistical analyses

All statistical analyses were performed using SPSS Statistics (version 23.0, IBM; Armonk, NY). Graphs were conducted with R software (version 4.0.2, R Foundation). The normality of distributions of continuous variables was checked by the Kolmogorov-Smirnov test. Data that were not normally distributed are expressed as medians and interquartile ranges (IQRs). Categorical variables are presented as numbers and percentages. Comparisons between groups were analysed using the Mann-Whitney *U* test, *χ*^*2*^ test, or Fisher’s exact test, as appropriate. Clinical features and 28-day all-cause mortality during hospitalisation were analysed and compared between patients with and without diabetes. Cumulative survival rates were plotted by the Kaplan-Meier method with the log-rank test. Risk factors associated with in-hospital death and their corresponding hazard ratios (HRs) and 95% confidence intervals (CIs) were analysed using univariable and multivariable Cox regression analyses (likelihood ratio method). Sensitivity analysis was performed in a subgroup of patients with HbA1c results at admission, and risk factors for in-hospital death were also evaluated with logistic regression analysis. A two-sided *P* value < 0.05 was considered statistically significant.

## Results

### Clinical characteristics of patients at admission

Of the 373 patients with COVID-19, 101 patients transferred from Fangcang hospitals were excluded. An additional 39 patients were excluded because of missing key variables (30 cases), end-stage renal disease (six cases), renal transplantation (two cases), and systemic lupus erythematosus under continuous corticosteroid therapy (one case). In total, 233 patients were included in the final analysis. Eighty (34.3%) patients had diabetes, among whom 44 (55.0%) were previously diagnosed and 36 (45.0%) were newly defined as having undiagnosed diabetes with an HbA1c level ≥ 6.5% (48 mmol/mol) at admission (Fig. 1). All of them were classified as having type 2 diabetes (T2D) based on the physicians’ clinical evaluation. One middle-aged woman with undiagnosed diabetes developed diabetic ketoacidosis on admission. She achieved well controlled glucose level with oral antidiabetic drugs after receiving intensive insulin therapy and supportive treatment for COVID-19.

The demographic and clinical characteristics and laboratory findings of patients with COVID-19 at admission are presented in Table 1. The median age was 64 years, and there were 115 (49.4%) males. The most common comorbidities other than diabetes were hypertension (90 cases, 38.6%), coronary artery disease (26 cases, 11.2%), and cerebrovascular disease (12 cases, 5.2%). At admission, 115 (49.4%), 95 (40.8%), and 23 (9.8%) patients were classified as moderate, severe, and critical cases, respectively.

**Table 1:** Demographic, clinical, and laboratory characteristics at admission, and in-hospital death in patients with COVID-19

	Total (*n* = 233)	Non-diabetes (*n* = 153)	Diabetes (*n* = 80)	Diabetes vs. Non-diabetes	***χ2 or Z***	***p*** value
**Demographic and clinical characteristics**						
Age (years)	64.0 (52.0–71.0)	64.0 (47.0–69.5)	66.0 (58.0–72.0)	2.0	2.530	0.011
Sex					0.175	0.676
Male	115 (49.4%)	74 (48.4%)	41 (51.3%)	2.9%		
Female	118 (50.6%)	79 (51.6%)	39 (48.7%)	−2.9%		
Comorbidities						
Hypertension	90 (38.6%)	47 (30.7%)	43 (53.8%)	23.1%	11.754	0.001
Coronary artery disease	26 (11.1%)	12 (7.8%)	14 (17.5%)	9.7%	4.942	0.026
Cerebrovascular disease	12 (5.2%)	5 (3.3%)	7 (8.8%)	5.5%	3.232	0.072
Chronic pulmonary disease	20 (8.6%)	12 (7.8%)	8 (10.0%)	2.2%	0.311	0.577
qSOFA score	0 (0–1)	0 (0–1)	1 (0–1)	1	1.632	0.103
Systolic blood pressure ≤ 100 mmHg	15 (6.4%)	8 (5.2%)	7 (8.8%)	3.6%	1.081	0.298
Respiratory rate ≥ 22 breaths per min	102 (43.8%)	63 (41.2%)	39 (48.8%)	7.6%	1.224	0.269
Altered mentation	6 (2.6%)	2 (1.3%)	4 (5.0%)	3.7%	–	0.185
Disease severity classification at admission					18.644	< 0.001
Moderate	115 (49.4%)	89 (58.2%)	26 (32.5%)	−25.7%		
Severe	95 (40.8%)	56 (36.6%)	39 (48.8%)	12.2%		
Critical	23 (9.9%)	8 (5.2%)	15 (18.8%)	13.6%		
**Laboratory findings**						
White blood cell count (× 10^9^/L)	5.3 (4.3–7.2)	5.1 (4.1–6.2)	6.1 (4.9–9.1)	1.0	3.809	< 0.001
Lymphocyte count (× 10^9^/L)	1.0 (0.7–1.4)	1.1 (0.7–1.5)	0.9 (0.6–1.3)	−0.2	2.100	0.036
Alanine aminotransferase (U/L)	21 (14–39)	20 (14–36)	24 (16–43)	4	1.492	0.136
Creatinine (μmol/L)	70 (57–85)	68 (55–82)	76 (61–94)	8	2.223	0.026
Fasting plasma glucose (mmol/L)	5.8 (5.3–7.1)	5.5 (5.1–6.1)	7.5 (6.3–11.2)	2.0	8.527	< 0.001
HbA1c (%)^a^	6.4 (5.9–7.3)	6.0 (5.7–6.2)	7.2 (6.6–8.6)	1.2	9.563	< 0.001
HbA1c (mmol/mol) ^a^	46 (41–56)	42 (39–44)	55 (49–70)	13	9.563	< 0.001
Interleukin-6 ≥ 13.26 pg/mL^b^	114/228 (50.0%)	69/148 (46.6%)	45/80 (56.3%)	9.7%	1.926	0.165
D-dimer ≥1 μg/mL^b^	114/229 (49.8%)	63/149 (42.3%)	51/80 (63.7%)	21.4%	9.596	0.002
**In-hospital death**	27 (11.6%)	9 (5.9%)	18 (22.5%)	16.6%	14.159	< 0.001

Data are expressed as median (interquartile range) or n (%) as appropriate. ^a^ Analysed in 140 cases with HbA1c data. *n* = 70 in the non-diabetes group and n = 70 in the diabetes group. ^b^ Median value. *HbA1c* hemoglobin A1c, *qSOFA* quick sequential organ failure assessment

The median age was higher in patients with T2D than in those without diabetes, and patients with T2D also had a higher rate of pre-existing hypertension and coronary artery disease. More patients in the diabetes group were classified as having severe and critical cases than those in the non-diabetes group. No significant difference was found between groups in sex, other comorbidities, or qSOFA score at admission. Patients with T2D had higher fasting plasma glucose and HbA1c levels, higher white blood cell counts, lower lymphocyte counts, and higher serum creatinine and D-dimer levels at admission than patiens without diabetes (Table 1).

In the subgroup analysis, patients with undiagnosed diabetes had more comorbid chronic pulmonary diseases, higher qSOFA scores, more severe and critical cases, higher fasting plasma glucose and HbA1c levels, higher white blood cell counts, and higher serum creatinine and D-dimer levels at admission than those without diabetes (Additional Table 1).

### In-hospital mortality rate

**Fig. 2:**
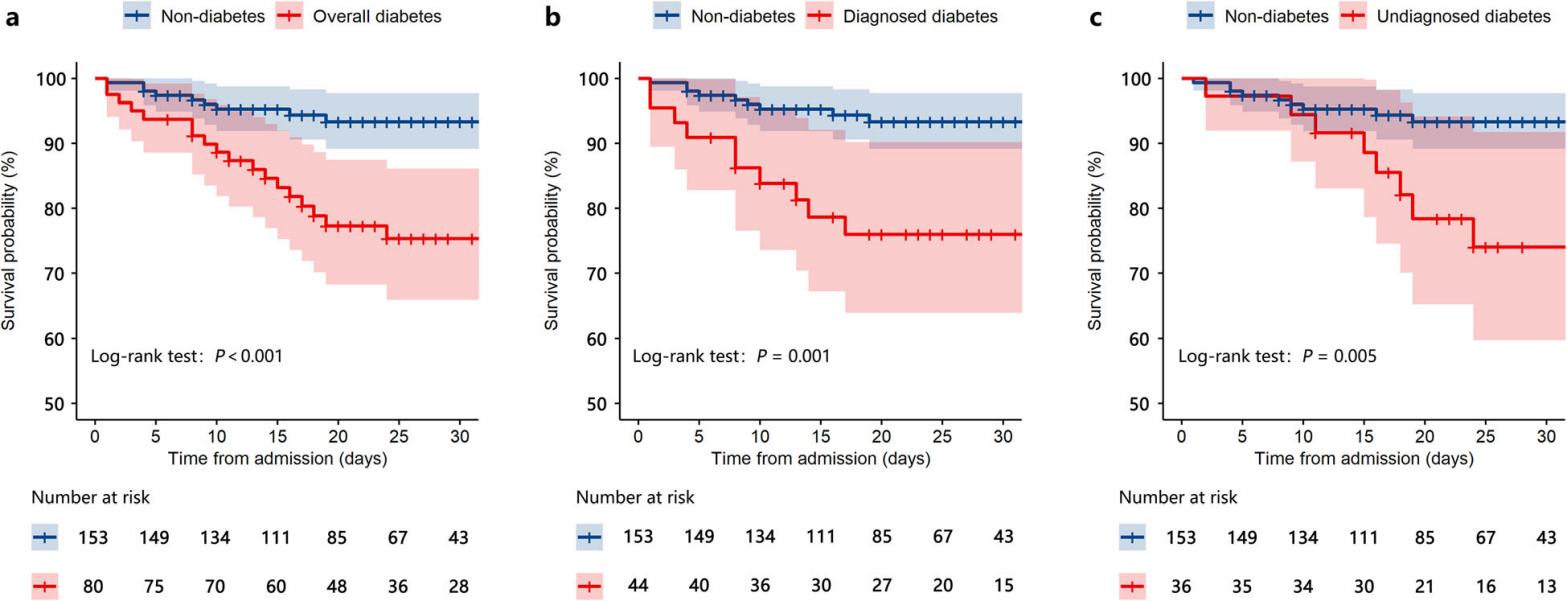
Survival probability of inpatients with COVID-19. Kaplan-Meier survival curves of patients with COVID-19 belonging to the overall diabetes (**a**), diagnosed diabetes (**b**), and undiagnosed diabetes (**c**) groups versus that of patients in the non-diabetes group. The blue and pink areas represent 95% CIs

Twenty-seven patient deaths occurred during hospitalisation, all within 28 days after admission. The in-hospital mortality rate was higher in the overall (22.5% vs. 5.9%, *p* <  0.001), diagnosed (22.7% vs. 5.9%, *p* = 0.001), and undiagnosed diabetes (22.2% vs. 5.9%, *p* = 0.002) groups than in the non-diabetes group (Table 1, Additional Table 1). The mortality rate did not significantly differ between patients with undiagnosed and diagnosed diabetes (22.2% vs. 22.7%, *p* = 0.957). The survival curves of patients with or without diabetes are shown in Fig. 2a, indicating that the survival probability was lower in patients with diabetes than in those without. Moreover, the probability of survival was significantly decreased in patients with both diagnosed and undiagnosed diabetes compared to those without diabetes (Fig. 2b and c). In a subgroup of 140 patients who had their HbA1c level tested, the survival probability was still lower in patients in the overall, diagnosed, and undiagnosed diabetes groups than in the non-diabetes group (Additional Fig. 1).

### Risk factors associated with in-hospital death in patients with COVID-19

To further investigate whether diabetes was independently associated with an increased risk of mortality in patients with COVID-19, Cox regression analysis was performed. Using univariable analysis, it was found that the risk of in-hospital death was significantly increased in all patients with diabetes (HR 3.80, 95% CI 1.71–8.47), those with diagnosed diabetes (HR 4.03, 95% CI 1.64–9.91), and those with undiagnosed diabetes who were newly defined by HbA1c testing at admission (HR 1.89, 95% CI 1.18–3.05) compared to those without diabetes. Age, qSOFA score, white blood cell count, lymphocyte count, fasting plasma glucose level, and D-dimer level ≥ 1 μg/mL at admission were also significantly associated with the risk of in-hospital death (Table 2). Additionally, patients with high HbA1c (≥ 6.5%) or fasting glucose level (≥ 7.0 mmol/L) at admission had increased mortality risk (Table 3).

**Table 2:** Risk factors associated with in-hospital death in patients with COVID-19 by Cox regression analysis

Variables	Univariable HR (95% CI)	***p*** value	Multivariable HR (95% CI)	***p*** value
**Demographic and clinical characteristics**				
Age (years)	1.08 (1.04–1.12)	< 0.001	1.07 (1.02–1.10)	0.001
Sex-male	2.12 (0.95–4.72)	0.066	–	0.052
Diabetes	3.80 (1.71–8.47)	0.001	2.64 (1.14–6.11)	0.024
Diagnosed	4.03 (1.64–9.91)	0.002	–	–
Undiagnosed	1.89 (1.18–3.05)	0.009	–	–
Hypertension	1.45 (0.68–3.08)	0.339	–	–
Coronary artery disease	1.05 (0.32–3.49)	0.935	–	–
Cerebrovascular disease	2.68 (1.81–8.90)	0.108	–	–
Chronic pulmonary disease	2.54 (0.96–6.71)	0.060	–	0.134
qSOFA score	2.86 (1.68–4.87)	< 0.001	2.80 (1.58–4.97)	0.001
**Laboratory findings at admission**				
White blood cell count (×10^9^/L)	1.19 (1.12–1.25)	< 0.001	–	–
Lymphocyte count (×10^9^/L)	0.29 (0.11–0.77)	0.013	–	0.351
Alanine aminotransferase (U/L)	1.00 (0.99–1.01)	0.838	–	–
Creatinine (μmol/L)	1.00 (0.99–1.01)	0.112	–	–
Fasting plasma glucose (mmol/L)	1.14 (1.07–1.21)	< 0.001	–	–
HbA1c (%)	1.09 (0.81–1.45)	0.577	–	–
Interleukin-6 ≥ 13.26 pg/mL^a^	1.08 (0.49–2.36)	0.856	–	–
D-dimer ≥1 μg/mL^a^	5.77 (1.99–16.69)	0.001	3.28 (1.12–9.64)	0.030

^a^ Median value. *HbA1c* hemoglobin A1c, *HR* hazard ratio, *qSOFA* quick sequential organ failure assessment

**Table 3:** Glycaemic level at admission and the risk of in-hospital death in patients with COVID-19

Variable	Number	In-hospital death n (%)	Univariable HR (95% CI)	***p*** value
**HbA1c (%)**				
≤ 5.6	15	0 (0)	–	–
5.7 ~ 6.4	57	2 (3.5)	Reference	–
≥ 6.5	68	14 (20.6)	5.80 (1.32, 25.53)	0.020
**Fasting plasma glucose (mmol/L)**				
≤ 5.5	87	2 (2.3)	Reference	–
5.6 ~ 6.9	82	7 (8.5)	3.49 (0.73, 16.82)	0.119
≥ 7.0	64	18 (28.1)	12.64 (2.93, 54.48)	0.001

*HR* hazard ratio*, HbA1c* hemoglobin A1c

Subsequently, we used age, diabetes, qSOFA score, lymphocyte count, and high D-dimer level as variables for multivariable Cox regression analysis. In addition, male sex and chronic pulmonary disease, which both reached 10% significance in the univariable analysis, were also included. A total of 223 patients with complete data for all analysed variables were included in the multivariable Cox regression model. Age (HR 1.07, 95% CI 1.02–1.10), diabetes (HR 2.64, 95% CI 1.14–6.11), qSOFA score (HR 2.80, 95% CI 1.58–4.97), and D-dimer level ≥ 1 μg/mL (HR 3.28, 95% CI 1.12–9.64) at admission were independently associated with an increased risk of in-hospital death in patients with COVID-19 (Table 2). Results of the multivariable logistic regression analysis were consistent with those of the Cox regression analysis (Additional Table 2).

## Discussion

In this retrospective observational study, the prevalence of diabetes in patients with non-mild COVID-19 cases was 34.3%. Among the patients with T2D, 45.0% were unaware of their underlying diabetes condition before admission. Diabetes was independently associated with an increased risk of in-hospital death in patients with COVID-19. Notably, patients with undiagnosed diabetes who were newly defined by HbA1c testing at admission had an increased risk of mortality during hospitalisation similar to that of patients with diagnosed diabetes, compared with their non-diabetes counterparts.

Diabetes has been garnering attention in terms of its prevalence and impact in the COVID-19 population. A report on the largest case series of COVID-19 in China, conducted by the Chinese National Emergency Response Epidemiology Team, showed that the prevalence of diabetes among 44,672 confirmed Chinese mainland patients with COVID-19 was 5.3% [20]. Observational studies and meta-analyses reported that the prevalence of pre-existing diabetes in Chinese patients with COVID-19 ranged from 8.2 to 19.0% [8, 21–23]. Here, we showed a much higher prevalence of diabetes (34.3%) in patients with COVID-19. This could be due to two reasons. First, our patients were from one of the national intensive care centres for COVID-19 that only admitted moderate to critical patients. The patients in our study were older and had more severe conditions than those in the nationwide analysis [20, 21]. Therefore, a higher prevalence of diabetes was expected in this study, similar to that reported by medical centres in Western countries [6, 11, 24, 25]. This might also suggest an association between pre-existing diabetes and an increased severity of COVID-19. Second, we included patients with newly diagnosed diabetes defined by HbA1c testing at admission. By contrast, most previous studies reported the prevalence of diabetes as a comorbidity according to patient histories of those with COVID-19, and patients who were included in non-diabetes groups had no available HbA1c data [23] or some of them had HbA1c levels over 6.5% [10]. In the most recent national epidemiological survey involving 75,880 adult participants, the prevalence of overall, self-reported, and newly diagnosed diabetes based on the American Diabetes Association criteria were 12.8, 6.0, and 6.8%, respectively, in China [26]. In agreement with that study, we found that approximately 50% of patients with T2D (elevated HbA1c levels) were undiagnosed before admission. HbA1c was first introduced into the American Diabetes Association diagnostic criteria of diabetes in 2010 [27]. HbA1c testing can well represent average blood glucose levels within 2–3 months before testing and is not influenced by factors such as acute infection, stress, or recent medications that could alter glucose metabolism, like corticosteroids. Moreover, HbA1c testing does not require fasting. Therefore, HbA1c is a reasonable diagnostic parameter for the quick identification of the background glucose metabolic state in severe and critical patients with COVID-19. Because diabetes is one of the most common comorbidities in patients with COVID-19 and is associated with poor outcomes, HbA1c testing at admission can provide important information for patient assessment and help identify those who have not been diagnosed but are at great risk.

It has been shown that patients with diabetes have poorer COVID-19 outcomes. The prevalence of diabetes is much higher in patients with COVID-19 treated in intensive care units than in those treated in general wards [5]. Patients with diabetes had a higher risk of developing severe or critical COVID-19 [23] and having multiple-organ damage, and a higher mortality rate than patients without diabetes [10, 11, 14, 15, 20]. The risk of COVID-19 hospitalisation was elevated in community people with poorly controlled diagnosed diabetes, and even in those with undiagnosed diabetes (HbA1c ≥ 6.5% at baseline) [28]. It is not surprising that both higher HbA1c and fasting glucose levels at admission were associated with higher mortality rate which were found by our and previous studies [29, 30]. Similar to previous studies [8, 21], our data indicated that diabetes, together with advanced age, a high qSOFA score, and coagulation disorders, was a risk factor for in-hospital death in moderate to critical patients with COVID-19. Similarly, diabetes was also previously reported as a major risk factor for mortality in severe acute respiratory syndrome in 2003 and Middle East respiratory syndrome [31, 32]. Thus far, there is no established effective therapy for reducing the mortality rate of COVID-19. However, a recent study reported that a well-controlled blood glucose level in patients with diabetes during hospitalisation was associated with a markedly reduced mortality from COVID-19, in comparison with poorly controlled glycaemia [10]. Therefore, identifying undiagnosed diabetes provides awareness of the background glycaemic disorder, thereby facilitating appropriate intervention for at-risk patients with coronavirus infections, including glucose monitoring and glycaemic control, and possibly better outcomes.

The underlying mechanism of the impact of diabetes on the prognosis of COVID-19 is still under investigation. It is well known that patients with diabetes are usually older and have more complications or comorbidities than those without diabetes. Increased number of underlying cardiometabolic conditions (e.g., diabetes, hypertension, and dyslipidaemia) is associated with higher mortality rate in patients with COVID-19 [33]. Notably, in line with our findings, several studies also showed that diabetes was an independent risk factor for poor prognosis in patients with COVID-19 after adjusted for the confounders [10, 28, 33], suggesting that diabetes itself, under its multiple mechanisms, may have adverse effects on the prognosis of COVID-19.

The dysregulated immune response caused by diabetes may contribute to increased disease severity. COVID-19 patients with diabetes have more neutrophils and a higher rate of lymphopenia [10], which is in agreement with our findings of higher white blood cell counts and lower lymphocyte counts in patients with T2D than in those without diabetes. In addition, diabetes may cause a chronic inflammatory state, elevating the levels of pro-inflammatory cytokines, such as interleukin-1 (IL-1) and IL-6, and further aggravate cytokine storms in some patients with COVID-19 [34–36]. Accordingly, both higher HbA1c and fasting glucose levels at admission, two indicators for poor glycaemic control, are associated with excessive inflammation and hypercoagulability in patients with COVID-19 [29, 30]. Furthermore, in hyperglycaemic patients, higher plasma IL-6 levels were associated with reduced effects of tocilizumab [37], indicating that hyperglycaemia may cause exaggerative and harmful inflammation in patients with COVID-19. However, our study did not show a significant difference in serum IL-6 levels between groups.

Angiotensin-converting enzyme 2 (ACE2) may be another underlying mechanism for the detrimental effects of diabetes on the prognosis of COVID-19. SARS-CoV-2 gains entry into host pneumocytes by binding to ACE2 [38]. Patients with diabetes were reported to have a higher expression of ACE2, thereby facilitating viral uptake and increasing the risk of severe infection [39]. Moreover, glucose can also directly increase the viral load and upregulate the expression of ACE2 and IL-1β in SARS-CoV-2-infected monocytes in a dose-dependent manner, suggesting that individuals with elevated circulating glucose levels may be more susceptible to SARS-CoV-2 infection and more likely to develop severe illness [40]. We noticed that patients with T2D had a higher rate of pre-existing hypertension in our study, which may bring concerns of possible effects by ACE2 inhibitors (ACEIs) and angiotensin receptor blockers (ARBs) on the prognosis of COVID-19. However, there is no evidence that ACEIs or ARBs could increase the expression of ACE2 in human beings [41]. Importantly, there is no clinically significantly increased risk of COVID-19 diagnosis or hospital admission-related outcomes associated with ACEIs/ARBs use in prospective and retrospective cohort studies [42–44]. Therefore, we are not worried that ACEIs/ARBs exposure may change our findings substantially.

Our study has several advantages. Our study focused on the clinical outcomes of both undiagnosed and diagnosed diabetes in patients with COVID-19. The HbA1c determination method used in our centre is comparable to the National Glycohemoglobin Standardisation Programme standard. By testing the HbA1c level at admission, we reduced the omission diagnostic rate of diabetes and prevented the overdiagnosis of diabetes because of stress-induced hyperglycaemia. The high percentage of undiagnosed diabetes, together with the similarly worse clinical outcome of undiagnosed and diagnosed diabetes compared with non-diabetes, highlighted the importance of screening for undiagnosed diabetes by HbA1c detection in patients with COVID-19. Moreover, the patients included in this study were admitted at a single medical centre and underwent treatments following uniform clinical guidelines, thereby reducing bias resulting from different treatment methods. Finally, we presented survival curves of COVID-19 patients with and without diabetes, while most previous studies only showed final outcomes without time-kinetic changes. Shi et al. [23] reported survival curves of patients with COVID-19, in which the survival probability of patients with diabetes was lower than that of sex- and age-matched patients without diabetes. However, in their study, patients in the non-diabetes control group had no available HbA1c data, and the fasting glucose levels in some cases were over 11.1 mmol/L, indicating a high possibility of patients with undiagnosed diabetes in the control group.

Our study has some limitations. First, it was a single-centre study with a limited number of patients. We enrolled as many patients as we could and excluded patients who were transferred from Fangcang hospitals to reduce bias from pre-admission treatment. Second, not all patients had their HbA1c level tested during the hospitalisation, particularly those in the non-diabetes group, as not all medical teams in our COVID-19 wards had members specialising in endocrinology. At the very beginning of the pandemic in Wuhan, some medical staff had not realised the potential benefit of evaluating and managing glucose metabolism in patients with COVID-19. Therefore, some patients did not undergo HbA1c testing; thus, the prevalence of diabetes in our COVID-19 population may even be higher than what we reported in this study. Nevertheless, in the subgroup analysis of 140 patients with available HbA1c data, the association between lower survival probability and diabetes (overall, diagnosed, or undiagnosed diabetes) was consistent with the results of the primary analysis of all 233 patients. Third, we only included IL-6 in the risk factor analysis and did not analyse other inflammatory biomarkers, such as serum C-reactive protein (CRP) or ferritin, in the present study. Most patients were tested for high-sensitivity CRP (hsCRP), rather than CRP, because hsCRP was incorporated into the biochemical analysis at our medical centre. hsCRP levels are more associated with systemic low-grade inflammation than with acute inflammatory conditions, such as COVID-19. In addition, IL-6, which is upstream of CRP as a sensitive marker for acute infection, was tested in most of our patients at admission. Therefore, we used IL-6 as the inflammatory biomarker in our Cox regression analysis. Although ferritin data were available in 223 patients, many were tested several days after admission, indicating that the levels could be confounded by other in-hospital factors. Thus, ferritin was excluded in the final analysis.

## Conclusion

The prevalence of diabetes is high (34.3%) in adult patients with non-mild COVID-19 cases in China, with 45.0% of the patients being unaware of their underlying diabetes condition. Importantly, similar to patients with diagnosed diabetes, patients with undiagnosed diabetes are also at a higher risk of in-hospital death from COVID-19. Therefore, HbA1c testing should be considered for all adult inpatients with COVID-19 to help clinicians identify patients with undiagnosed diabetes and provide appropriate management for this potentially high-risk population, including glucose monitoring and glycaemic control, in order to achieve better outcomes.

## Supplementary Information

**Additional file 1: Figure S1.** Survival probability of inpatients with COVID-19 who underwent HbA1c testing. Kaplan-Meier survival curves of patients with COVID-19 belonging to the overall diabetes (a), diagnosed diabetes (b), and undiagnosed diabetes (c) groups versus that of patients without diabetes in a subgroup of participants who had their HbA1c tested at admission. The blue and pink areas represent 95% CIs. *HbA1c* hemoglobin A1c.

## Data Availability

The datasets used and/or analysed during the current study are available from the corresponding author on reasonable request.

## Abbreviations

ACE: Angiotensin-converting enzyme
CI: Confident interval
COVID-19: Coronavirus disease 2019
CRP: C-Reactive protein
hcCRP: High-sensitivity CRP
HbA1c: Hemoglobin A1c
HR: Hazard ratio
IL: Interleukin
IQR: Interquartile range
qSOFA: quick sequential organ failure assessment
SARS-CoV-2: Severe acute respiratory syndrome coronavirus 2
T2D: Type 2 diabetes

## Declarations

### Data included in other reports

53 patients among the 373 patients in this study has been included in our previous research on the family companion between patients with COVID-19 [45]. No data about their glucose level has been reported previously.
