# Supplementary material for: Diabetes, even newly defined by HbA1c testing, is associated with an increased risk of in-hospital death in adults with COVID-19
Source: BMC Endocr Disord. 2021 Mar 26;21:56. doi: 10.1186/s12902-021-00717-6 (PMC7995677; doi:10.1186/s12902-021-00717-6)
Supplement: Supplementary file 1 — Additional file 1: Figure S1. Survival probability of inpatients with COVID-19 who underwent HbA1c testing. Kaplan-Meier survival curves of patients with COVID-19 belonging to the overall diabetes (a), diagnosed diabetes (b), and undiagnosed diabetes (c) groups versus that of patients without diabetes in a subgroup of participants who had their HbA1c tested at admission. The blue and pink areas represent 95% CIs. HbA1c hemoglobin A1c. [file 12902_2021_717_MOESM1_ESM.pdf]

Additional Table 1 Characteristics at admission, and in-hospital death in patients with COVID-19 belonging to diagnosed or undiagnosed diabetes versus those without diabetes

	**Non-diabetes (n=153)**	**Diagnosed diabetes (n=44)**	***p* value [a]**	**Undiagnosed diabetes (n=36)**	***p* value [b]**
**Demographic and clinical characteristics**					
Age (years)	64.0 (47.0–69.5)	66.0 (60.2–72.0)	0.006	65.0 (55.5–71.5)	0.287
Sex					
Male	74 (48.4%)	23 (52.3%)	0.648	18 (50.0%)	0.860
Female	79 (51.6%)	21 (47.7%)		18 (50.0%)	
Comorbidities					
Hypertension	47 (30.7%)	29 (65.9%)	< 0.001	14 (38.9%)	0.345
Coronary artery disease	12 (7.8%)	13 (29.5%)	< 0.001	1 (2.8%)	0.468
Cerebrovascular disease	5 (3.3%)	5 (11.4%)	0.031	2 (5.6%)	0.620
Chronic pulmonary disease	12 (7.8%)	1 (2.3%)	0.304	7 (19.4%)	0.037
qSOFA score	0 (0–1)	0 (0–1)	0.515	1 (0– 1)	0.048
Disease severity classification at admission			0.010		< 0.001
Moderate	89 (58.2%)	15 (34.1%)		11 (30.6%)	
Severe	56 (36.6%)	23 (52.3%)		16 (44.4%)	
Critical	8 (5.2%)	6 (13.6%)		9 (25.0%)	
**Laboratory findings**					
White blood cell count ($\times10^9$/L)	5.1 (4.1–6.2)	5.8 (4.7–7.1)	0.045	7.6 (5.0–10.6)	< 0.001
Lymphocyte count ($\times10^9$/L)	1.1 (0.7–1.5)	0.85 (0.6–1.2)	0.041	0.92 (0.5–1.4)	0.241
Alanine aminotransferase (U/L)	20 (14–36)	22 (16–37)	0.458	25 (15–46)	0.103
Creatinine (μmol/L)	68 (55–82)	75 (61–93)	0.160	79 (60–97)	0.036
Fasting plasma glucose (mmol/L)	5.5 (5.1–6.1)	8.0 (6.5–10.8)	< 0.001	7.1 (5.9–12.2)	< 0.001
HbA_{1c} (%)[c]	6.0 (5.7–6.2)	7.9 (7.2–8.8)	< 0.001	6.8 (6.5–8.1)	< 0.001
HbA_{1c} (mmol/mol)[c]	42 (39–44)	63 (55–73)	< 0.001	51 (48–65)	< 0.001
Interleukin-6 ≥ 13.26 pg/mL[d]	69/148 (46.6%)	25/44 (56.8%)	0.235	20/36 (55.6%)	0.336
D-dimer ≥ 1 μg/mL[d]	63/149 (42.3%)	27/44 (61.4%)	0.026	24/36 (66.7%)	0.009
**In-hospital death**	9 (5.9%)	10 (22.7%)	0.001	8 (22.2%)	0.002

Data are expressed as median (interquartile range) or n (%) as appropriate. [a] Diagnosed diabetes *vs* non-diabetes. [b] Undiagnosed diabetes *vs* non-diabetes. [c] Analysed in 140 cases having HbA_{1c} data. n=70, 34 and 36 in non-diabetes, diagnosed diabetes and undiagnosed diabetes groups, respectively. [d] Median value.

HbA1c hemoglobin A1c, *qSOFA* quick sequential organ failure assessment.

Additional Table 2 Risk factors associated with in-hospital death in patients with COVID-19 by logistic regression analysis

Variables	Univariable OR (95% CI)	*p* value	Multivariable OR (95% CI)	*p* value
**Demographic and clinical characteristics**				
Age (years)	1.08 (1.04–1.12)	< 0.001	1.07 (1.02–1.12)	0.004
Sex–male	2.25 (0.97–5.24)	0.061	–	–
Diabetes	4.65 (1.98–10.91)	< 0.001	3.99 (1.48–10.82)	0.006
Diagnosed	4.71 (1.78–12.48)	0.002	–	–
Undiagnosed	4.57 (1.62–12.87)	0.004	–	–
Hypertension	1.56 (0.70–3.48)	0.283	–	–
Coronary artery disease	1.00 (0.28–3.56)	0.993	–	–
Cerebrovascular disease	2.74 (0.69–10.81)	0.151	–	–
Chronic pulmonary disease	2.89 (1.00–8.73)	0.059	–	–
qSOFA score	3.52 (1.87–6.62)	< 0.001	3.35 (1.63–6.87)	0.001
**Laboratory findings at admission**				
White blood cell count ($\times 10^9$/L)	1.29 (1.17–1.44)	< 0.001	–	–
Lymphocyte count ($\times 10^9$/L)	0.24 (0.09–0.68)	0.007	–	0.399
Alanine aminotransferase (U/L)	1.00 (0.99–1.01)	0.757	–	–
Creatinine (μmol/L)	1.00 (1.00–1.01)	0.244	–	–
Fasting plasma glucose (mmol/L)	1.21 (1.10–1.33)	< 0.001	–	–
HbA_{1c} (%)	1.14 (0.83–1.57)	0.412	–	–
Interleukin-6 ≥ 13.26 pg/mL[a]	1.09 (0.48–2.51)	0.832	–	–
D-dimer ≥ 1 μg/mL[a]	7.01 (2.34–21.02)	0.001	3.42 (1.01–11.58)	0.048

[a] Median value.

HbA1c hemoglobin A1c, *OR* odds ratio, *qSOFA* quick sequential organ failure assessment.

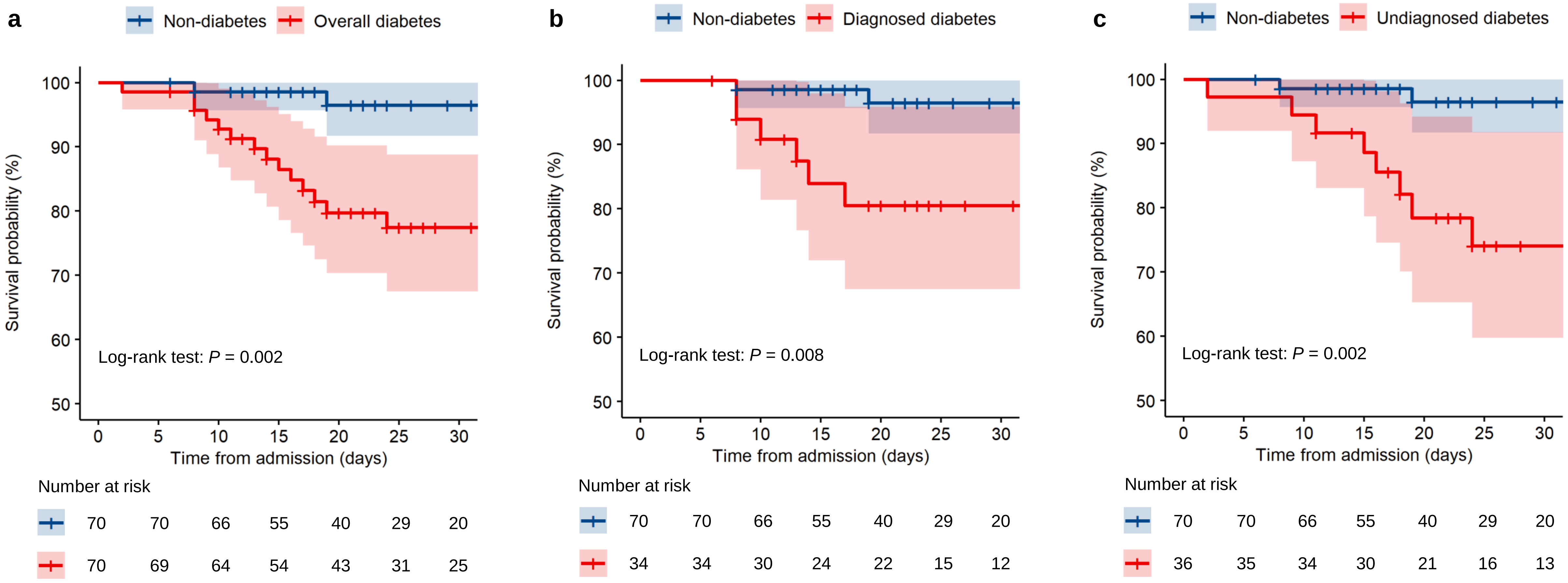

Additional Fig. 1 Survival probability of inpatients with COVID-19 who underwent HbA1c testing. Kaplan-Meier survival curves of patients with COVID-19 belonging to the overall diabetes (a), diagnosed diabetes (b), and undiagnosed diabetes (c) groups versus that of patients without diabetes in a subgroup of participants who had their HbA1c tested at admission. The blue and pink areas represent 95% CIs. *HbA1c* hemoglobin A1c.
